# Interplays of Dietary Fat with BMI and *FAAH* rs324420 on HDL‐C in Gender‐Dependent Manner in Adolescents

**DOI:** 10.1002/fsn3.4497

**Published:** 2024-10-03

**Authors:** Yi Lin Shen, Li Qiu, Jia Jing Cai, Qi Wei Guo, Xu Chen, Guo Ming Su, Jia Lin, Ding Zhi Fang

**Affiliations:** ^1^ Department of Biochemistry and Molecular Biology, West China School of Basic Medical Sciences and Forensic Medicine Sichuan University Chengdu China

**Keywords:** fatty acid amide hydrolase, gene polymorphism, intake of fat, serum lipids

## Abstract

The present study was to explore relationship between serum lipid profiles and the polymorphism of rs324420 at fatty acid amide hydrolase (FAAH) gene (*FAAH* rs324420) and its confounders in Chinese adolescents. Serum lipids, glucose, and insulin levels were assessed using routine methods in a cohort of 708 high school students. Dietary intake was investigated by 3‐day diet records, and intakes of protein, fat, and carbohydrate were calculated. *FAAH* rs324420 was genotyped by polymerase chain reaction‐restriction fragment length polymorphism technique and confirmed through DNA sequencing. In the whole study population, increased HDL‐C levels were observed in *FAAH* rs324420 CC homozygotes than those in A allele carriers. Body mass index (BMI), gender, intake of fat, and *FAAH* rs324420 were predictive factors of HDL‐C levels in the whole study population. Moreover, BMI and intake of fat were predictors of HDL‐C levels in male *FAAH* rs324420 A allele carriers and female CC homozygotes, but only BMI was predictor of HDL‐C in female A allele carriers and male CC homozygotes. These results demonstrate that there are mutual effects of dietary fat with gender, BMI, and *FAAH* rs324420 on HDL‐C levels, which pave a novel way to explore the heterogeneous relationship of serum lipid profiles with diets or *FAAH* rs324420 and provide a new perspective of precision dietary interventions of dyslipidemia, especially in adolescents.

## Graphical abstract

The interactions occurred between fatty acid amide hydrolase gene (*FAAH*) rs324420 and dietary patterns to influence serum HDL‐C levels. Meanwhile, the dietary intake of fat affected the association of *FAAH* rs324420 with HDL‐C, which was gender‐dependent.

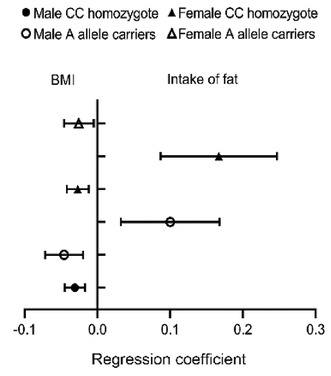

## 1 Introduction

The prevalence of metabolic syndrome (MetS) has been increasing rapidly not only in adults but also in adolescents (Shi et al. 2022; Zhu et al. 2020). In MetS, dyslipidemia is common, which mainly manifests as the reduction of high‐density lipoprotein cholesterol (HDL‐C), as well as increase of total cholesterol (TC), low‐density lipoprotein cholesterol (LDL‐C), and triglyceride (TG) (Mach et al. 2020). Although the mechanism of dyslipidemia has not been fully elucidated yet, abnormal serum lipid profiles are believed to be resulted from combined effects of genetic, environmental, behavioral, and developmental factors (Hannon, Khan, and Teran‐Garcia 2018; Matey‐Hernandez et al. 2018).

Serum lipid profiles are intensively affected by endocannabinoid system (ECS) (de Azua and Lutz 2019), in which fatty acid amide hydrolase (FAAH) is one of the key enzymes (Cravatt and Lichtman 2003; van Egmond, Straub, and van der Stelt 2021). FAAH plays a crucial role in the catabolism of arachidonoylethanolamine (anandamide, AEA) and, to a less extent, of 2‐arachidonoylglycerol (2‐AG) (Pamplona and Takahashi 2012). An elevation of FAAH gene (*FAAH*) mRNA was found in the subcutaneous abdominal adipose tissue from lean individuals when compared to that from obese individuals (Engeli et al. 2014; Murdolo et al. 2007). Interestingly, despite the observed increase in blood glucose levels and enhanced TG content in plasma, skeletal muscle, adipose tissue, and liver of *FAAH*‐deficient mice (Tourino et al. 2010), it was also demonstrated that hepatic cholesterol synthesis was decreased, and the levels of plasma TG remained unchanged in these mice (Vaitheesvaran et al. 2012). In addition, decreased levels of plasma glucose, TG, TC, and HDL‐C were observed in the rats treated with the FAAH inhibitor URB597 (Rivera et al. 2015).

*FAAH* rs324420 is a single‐nucleotide polymorphism (SNP) with the substitution from C to A at nucleotide 385 (C385A) of *FAAH*, resulting in a missense mutation from a proline residue to threonine residue at amino acid 129 (P129T) (Sipe et al. 2002). Consequently, it has been demonstrated that individuals with *FAAH* rs324420 AA homozygotes exhibit only half of FAAH protein expression and enzymatic activity compared to those with the wild‐type genotype (Chiang et al. 2004). Besides, *FAAH* rs324420 has been found to be linked with childhood obesity (Durand et al. 2008; Muller et al. 2010) and serum lipid profiles (Ning et al. 2017; Yagin et al. 2019). A previous systematic review, encompassing 65 articles and 70 SNPs, found an association between the mutation of *FAAH* rs324420 and elevated TG levels in European cohorts (Doris et al. 2019). Moreover, Zeng, Li, and Huang demonstrated that the A allele carriers exhibited higher serum TG levels and lower HDL‐C levels compared with the wild type in the subjects diagnosed with MetS (Zeng, Li, and Huang 2011). However, in 70 patients who developed diabetes mellitus type 2, higher BMI, weight, waist circumference, fat mass, and insulin levels were observed in the A allele carriers, but no striking discrepancies were found in serum TG, TC, HDL‐C, and LDL‐C between allelic groups (de Luis et al. 2010). The mechanism of these heterogeneous associations between *FAAH* rs324420 and serum lipid profiles needs to be clarified.

Dietary fat has been one of the most persistent and challenging issues. On one hand, a high‐fat diet has long been associated with dyslipidemia, which is a contributing factor to cardiovascular disease (Forouhi et al. 2018). On the other hand, some studies demonstrated that high‐fat diet decreased the levels of serum free fatty acids (FFAs) (Shi et al. 2021) and even the risk of cardiovascular disease (Dehghan et al. 2017). The intake of fat was reported to be positively associated with the levels of TC, LDL‐C, and HDL‐C, and negatively with TG (Mente et al. 2017; Song, Song, and Song 2017). However, Jacobsen et al. failed to find such associations between fat intake and HDL‐C in adults with type 1 diabetes and type 2 diabetes (Jacobsen et al. 2020). Moreover, epidemiologic studies suggested that dyslipidemia could be resulted from interplays between genetic and environmental factors (Matey‐Hernandez et al. 2018; Stein, Ferrari, and Scolari 2019), such as dietary patterns in different races (Feeney et al. 2017; Kafyra et al. 2021; Zhang et al. 2019). Nevertheless, most of the above investigations were carried out in adults or senile populations. Much less efforts have been made in adolescents. To explain the heterogeneous relationship between diets and serum lipid profiles and further explore the prospective mechanism for the regulations of serum lipid levels, we hypothesized that there might be interactions among diets, gender, and *FAAH* rs324420, resulting in alterations of serum lipid profiles. Therefore, our research aimed to investigate the changes in serum lipid profiles and their relationships with dietary patterns among healthy Chinese adolescents with different genotypes of *FAAH* rs324420.

## 2 Material and Methods

### 2.1 Subjects

A total of 746 students were recruited through advertising in a boarding high school located in Chengdu, Sichuan Province, China. Anthropometric measurements, biochemical examinations as well as medical and dietary questionnaires were used to obtain the characteristics of the volunteers. The inclusion criteria for the students to participate in the study were understanding the procedures involved and providing written consents from the students themselves and their guardians. The criteria for exclusion were a history of cardiovascular or other chronic diseases, taking hormones or other drugs that influenced glucose and lipid metabolisms for the last 3 months, and providing questionnaires not completely finished. Finally, the analysis encompassed 708 Chinese Han volunteers with all the data and blood samples. The study was approved by the Human Research Ethics Committee of Sichuan University.

### 2.2 Anthropometric Measurements

Anthropometric measurements were performed from 6:30 to 8:30 a.m. before breakfast. Regular procedures were employed to measure height and weight. Body mass index (BMI) was computed by dividing the weight in kilograms by the square of the height in meters (kg/m^2^).

### 2.3 Biochemical Examinations

Blood samples were collected in veins after 12 h‐fasting. Serum levels of TG, TC, LDL‐C, HDL‐C, and glucose were analyzed by routine methods in the laboratory. Briefly, semi‐automated biochemistry analyzers were enzymatically used to measure serum TG, TC, and glucose. LDL‐C levels were assessed using the polyvinyl sulfate precipitation method with a semi‐automated biochemistry analyzer. HDL‐C levels were enzymatically determined following the precipitation of apolipoprotein B‐containing lipoproteins using phosphotungstic‐Mg^2+^. Insulin levels were measured using chemiluminescence immunoassays. Each variable was measured in triplicate. All measurements were conducted in triplicate, with both inter‐ and intra‐assay coefficients of variation being < 6%.

### 2.4 Dietary Intake

Dietary questionnaires based on 3‐day diet records were used to collect the dietary intake as described before (Zhu et al. 2014). Before starting, educations were made to instruct the students in their classroom to complete the questionnaires, which were coded confidentially. The intakes of protein, fat, and carbohydrate were calculated by the computer‐based data evaluation system that developed by the Department of Nutrition and Food Safety of the Chinese Center for Disease Control (Beijing, China) and expressed as kilocalories (Kcal). Total energy intake was the sum of the intake of protein, fat, and carbohydrate.

### 2.5 DNA Extraction and Genotyping

**FIGURE 1:**
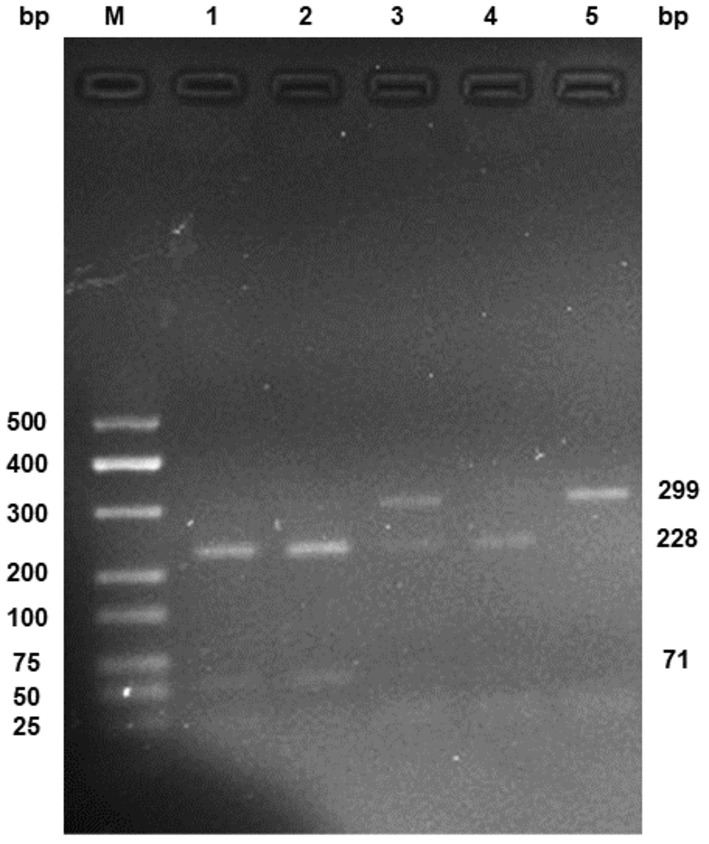
Electrophoretogram of PCR products containing *FAAH* rs324420 after digested by *Sty*I. M: DNA marker; 1, 2, 4: Fragments with CC genotype; 3: Fragments with CA genotype; 5: Fragments with AA genotype.

The DNAout kit (Cat No. 3671‐50, Tianze, China) was used to isolate the genomic DNA from peripheral blood leukocytes. Genotyping of *FAAH* rs324420 was carried out by polymerase chain reaction (PCR) using 5′‐GGAAGTGAACAAAGGGACCA‐3′ and 5′‐AGGGTCCACTCCAACAACTG‐3′ as forward and reverse primers, followed by restriction enzyme analyses. The cycling conditions for PCR were 95°C for 5 min followed by 35 cycles of 95°C for 30 s, 59°C for 30 s, and 72°C for 30 s and a final extension at 72°C for 5 min. The amplified DNA was subjected to the digestion by restriction enzyme *Sty*I (Cat No. R3500V, New England Biolabs, USA). Two microliters of PCR products were digested at 37°C overnight with 0.6 μL of *Sty*I enzyme in a final volume of 10 mL (Storr et al. 2009). The digested fragments were identified on gel electrophoresis of 2.5% agarose. The genotypes were identified by the resulting fragment patterns and verified by DNA sequencing. Specifically, the amplified fragments containing *FAAH* rs324420 site were 299 bp. As shown in Figure 1, the digestion of the PCR products with the AA genotype resulted in a single band of 299 bp fragments, the CC genotype in two bands of 228 bp and 71 bp fragments, and the CA genotype in three bands of 299 bp, 228 bp, and 71 bp fragments. These results were verified by DNA sequencing (Sangon Bioengineering Company, Shanghai, China).

### 2.6 Statistical Analyses

Data were presented as mean ± standard deviation (SD) besides its specification. Data normality was examined using Shapiro–Wilk tests. A chi‐square goodness‐of‐fit test was performed to examine whether the genotype distribution follows Hardy–Weinberg equilibrium. The chi‐square test was applied to analyze genotype and allele frequencies in individuals of different genders. Independent‐sample *t*‐tests were used to analyze the numerical variables of the subjects with different genotypes of *FAAH* rs324420. Factors influencing the levels of serum lipids, glucose, and insulin were determined by multiple linear regression analyses. The differences were judged statistically significant when *p* ≤ 0.05.

## 3 Results

### 3.1 Molecular Characterization of the Subjects

**FIGURE 2:**
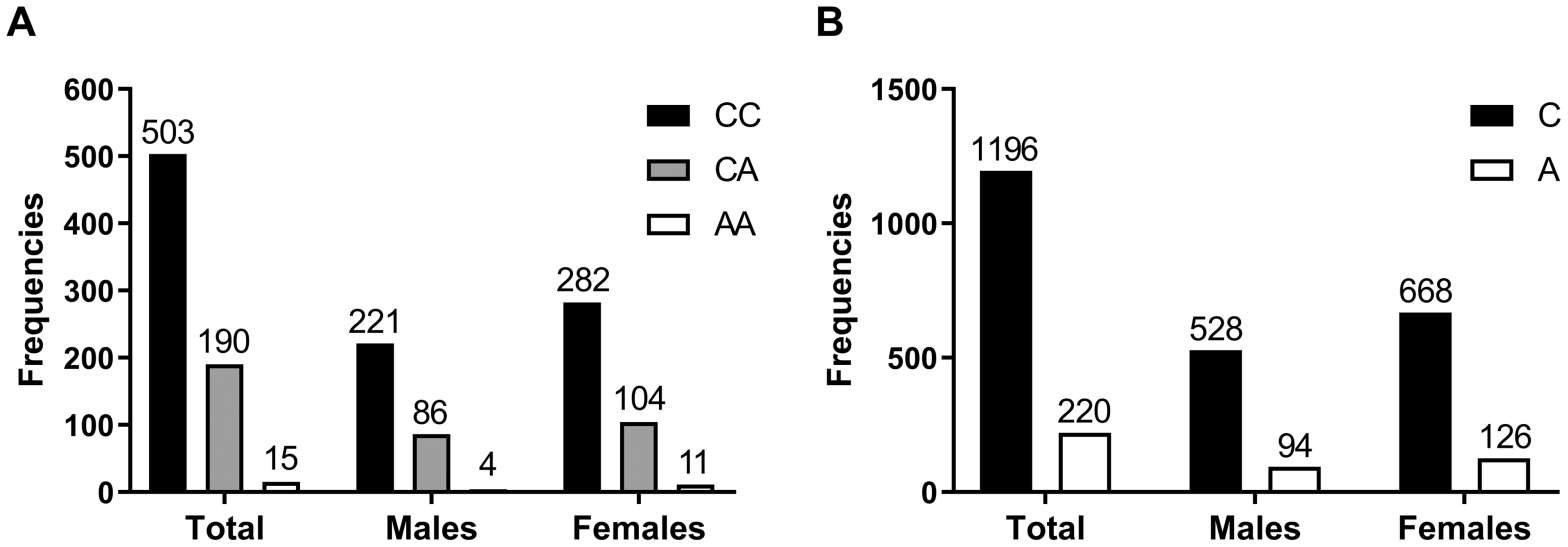
Frequencies of the genotypes and alleles of *FAAH* rs324420. No significant differences were observed in genotype and allele frequencies between the male and the female subjects by chi‐square tests.

Genotypes and allele frequencies of *FAAH* rs324420 in this research are displayed in Figure 2. The genotype distribution characteristics of *FAAH* rs324420 agree with Hardy–Weinberg equilibrium (*p* = 0.549). Due to the small total number of AA genotypes, which included 15 subjects, the individuals with the CA genotype and the AA genotype were jointly defined as the A allele carriers for further analyses. There were no distinct differences in the genotype (*p* = 0.377), and allele (*p* = 0.697) frequencies were observed between the male and female subjects.

### 3.2 Anthropometric, Biochemical, and Dietary Characteristics of the Subjects With Different *FAAH* rs324420 Genotypes

As indicated in Table 1, in the whole study population, only increased HDL‐C levels (*p* = 0.047) were observed in the CC homozygotes, when compared with those in the A allele carriers. On the contrary, there were no significant differences in the anthropometric and dietary characteristics between the CC homozygotes and the A allele carriers in the total study population.

**TABLE 1:** Anthropometric, biochemical, and dietary characteristics of the adolescents with different *FAAH* rs324420 genotypes.

Variables	Total (*n* = 708)	CC (*n* = 503)	AX (*n* = 205)
Males/females	311/397	221/282	90/115
Age, years	16.86 ± 0.59	16.86 ± 0.59	16.85 ± 0.58
BMI, kg/m^2^	20.29 ± 2.30	20.29 ± 2.33	20.29 ± 2.24
TG, mmol/L	1.12 ± 0.44	1.12 ± 0.46	1.10 ± 0.40
TC, mmol/L	3.59 ± 0.57	3.61 ± 0.59	3.53 ± 0.53
LDL‐C, mmol/L	1.67 ± 0.49	1.68 ± 0.50	1.64 ± 0.46
HDL‐C, mmol/L	1.41 ± 0.28	1.42 ± 0.29	1.38 ± 0.26^a^
Glucose, mmol/L	5.07 ± 0.44	5.06 ± 0.43	5.10 ± 0.46
Insulin, mIU/L	11.91 ± 5.59	11.73 ± 5.17	12.34 ± 6.49
Total energy intake, 10^3^ Kcal/day	2.73 ± 1.45	2.74 ± 1.44	2.73 ± 1.47
Intake of protein, 10^3^ Kcal/day	0.33 ± 0.21	0.33 ± 0.20	0.34 ± 0.24
Intake of fat, 10^3^ Kcal/day	0.90 ± 0.61	0.90 ± 0.60	0.90 ± 0.63
Intake of carbohydrate, 10^3^ Kcal/day	1.50 ± 0.73	1.51 ± 0.72	1.50 ± 0.74

Abbreviations: BMI, body mass index; FAAH, fatty acid amide hydrolase; HDL‐C, high‐density lipoprotein cholesterol; Kcal, kilocalorie; LDL‐C, low‐density lipoprotein cholesterol; TC, total cholesterol; TG, triglyceride.

^a^ *p* ≤ 0.05 when compared with those of the CC homozygotes (independent‐sample *t*‐tests).

### 3.3 Predictors of Serum Lipids, Glucose, and Insulin Levels

Multiple linear regression analyses were used to elucidate the factors influencing the serum levels of lipids, glucose and insulin, independent variables included BMI, age, gender, total energy intake, the intake of protein, the intake of fat, the intake of carbohydrate, and the genotype of *FAAH* rs324420, while the levels of serum lipids, glucose, and insulin were used as dependent variables separately. As presented in Table 2, the genotype of *FAAH* rs324420 was a predictor of only serum HDL‐C levels, but not serum glucose, insulin, TG, TC, and LDL‐C levels. In detail, BMI (*p* = 0.000), gender (*p* = 0.000), the intake of fat (*p* = 0.000), and the genotype of *FAAH* rs324420 (*p* = 0.045) were predictive factors of serum HDL‐C levels (Adjusted *R* ^2^ = 0.100).

**TABLE 2:** Predictors of serum lipids, glucose, and insulin levels in the study population.

Dependent variables	Independent variables	Adjusted *R* ^2^	*β*	95% CI	*p*
TG, mmol/L	BMI, kg/m^2^	0.154	0.045	0.032, 0.059	0.000
	Gender^a^		0.244	0.183, 0.305	0.000
TC, mmol/L	BMI, kg/m^2^	0.089	0.034	0.016, 0.052	0.000
	Gender^a^		0.285	0.202, 0.368	0.000
LDL‐C, mmol/L	BMI, kg/m^2^	0.063	0.046	0.031, 0.062	0.000
	Intake of carbohydrate, 10^3^ Kcal/day		−0.102	−0.150, −0.053	0.000
HDL‐C, mmol/L	BMI, kg/m^2^	0.100	−0.030	−0.039, −0.022	0.000
	Gender^a^		0.140	0.098, 0.181	0.000
	Intake of fat, 10^3^ Kcal/day		0.067	0.034, 0.101	0.000
	*FAAH* rs324420^b^		−0.044	−0.087, −0.001	0.045
Glucose, mmol/L	Age, years	0.070	−0.076	−0.129, −0.022	0.005
	Gender^a^		−0.114	−0.179, −0.048	0.001
	Intake of protein, 10^3^ Kcal/day		−0.336	−0.646, −0.027	0.033
	Intake of fat, 10^3^ Kcal/day		0.236	0.127, 0.345	0.000
Insulin, mIU/L	BMI, kg/m^2^	0.208	0.698	0.536, 0.860	0.000
	Gender^a^		3.471	2.718, 4.224	0.000

*Note:* BMI, age, gender, total energy intake, intake of protein, intake of fat, intake of carbohydrate, and FAAH rs324420 were included as independent variables (multiple linear regression analysis).

Abbreviations: BMI, body mass index; CI, confidence interval; FAAH, fatty acid amide hydrolase; HDL‐C, high‐density lipoprotein cholesterol; Kcal, kilocalorie; LDL‐C, low‐density lipoprotein cholesterol; TC, total cholesterol; TG, triglyceride; β, regression coefficient.

^a^ 0 = male, 1 = female.

^b^ 0 = CC, 1 = AX.

### 3.4 Predictors of Serum HDL‐C Levels in the Subjects With Different Genders and Genotypes of *FAAH* rs324420

**FIGURE 3:**
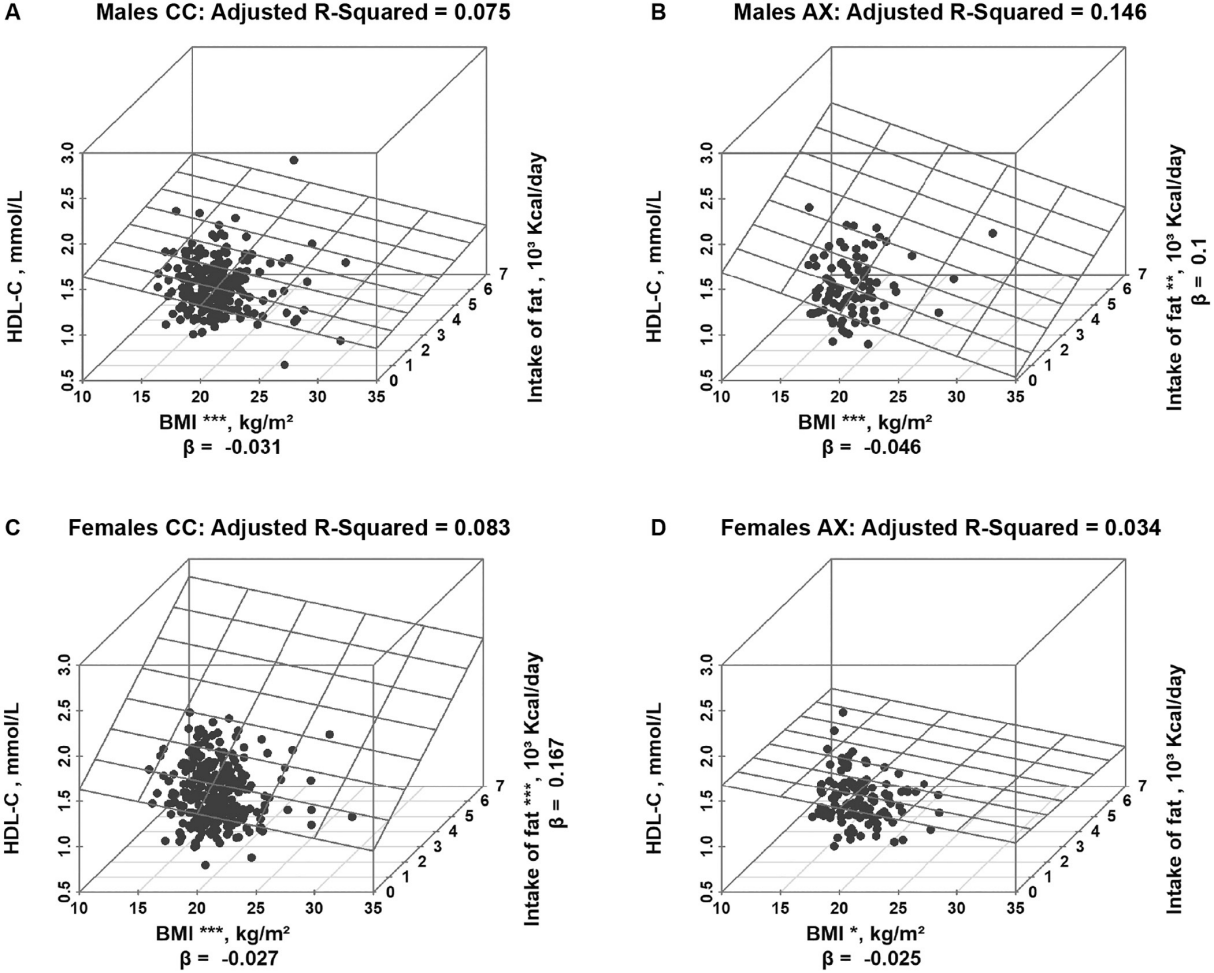
Predictors of serum HDL‐C levels in subjects with different genders and genotypes of *FAAH* rs324420. BMI, body mass index; FAAH, fatty acid amide hydrolase; HDL‐C, high‐density lipoprotein cholesterol; Kcal, kilocalorie; β, regression coefficient; *p ≤ 0.05, **p ≤ 0.01, ***p ≤ 0.001 (multiple linear regression analysis).

The above results showed that gender and genotype of *FAAH* rs324420, together with the intake of fat and BMI, had the potential to be predictors of serum HDL‐C levels. To further investigate the effect of dietary intake on HDL‐C, multiple linear regression analyses were performed in the male or the female individuals with different genotypes of *FAAH* rs324420 (Figure 3). Although BMI (*p* = 0.000 in the male CC homozygotes, *p* = 0.001 in the male A allele carriers, *p* = 0.000 in the female CC homozygotes, and *p* = 0.016 in the female A allele carriers) was a predictor regardless of the genders and the genotypes, the intake of fat (*p* = 0.004 in the male A allele carriers; *p* = 0.000 in the female CC homozygotes) was noticed to be a predictor of serum HDL‐C levels in the male A allele carriers (adjusted *R* ^2^ = 0.146) and the female CC homozygotes (adjusted *R* ^2^ = 0.083), but not in the female A allele carriers and the male CC homozygotes.

## 4 Discussion

Epidemiological studies have shown that there are relationships between ECS and serum lipid profiles (de Azua and Lutz 2019). FAAH, one of the key enzymes in this system (Cravatt and Lichtman 2003; van Egmond, Straub, and van der Stelt 2021), has been proven to be involved in lipid metabolism and associated with the levels of serum lipids (van Eenige et al. 2018). Therefore, exploring the above relationships may contribute to understand the regulation and its molecular mechanism of lipid metabolism and serum lipid levels, or even develop a promising strategy for the treatment of hyperlipidemia. However, there are conflicting reports about the correlation between *FAAH* rs324420 and serum lipid profiles (de Luis et al. 2010; Doris et al. 2019; Zeng, Li, and Huang 2011). Simultaneously, in the current research, significantly higher levels of HDL‐C were discovered in the CC homozygotes than in subjects with the A allele carriers in the whole study population (Table 1), which is inconsistent with the findings described in the previous study (de Luis et al. 2010; Zeng, Li, and Huang 2011). The endogenous cannabinoids, such as AEA and 2‐AG, have been proven to be related with food intake in both human and animal (Castonguay‐Paradis et al. 2020; van Ackern, Kuhla, and Kuhla 2021). Since FAAH is the main deactivating enzyme of AEA and 2‐AG, it may have the potential to be related to food intakes and even energy intakes of carbohydrate, fat, and protein. All the above suggested a gene–nutrient interaction to modulate serum lipid profiles.

Studies showed that the A allele of *FAAH* rs324420 was correlated with the significant reduction in serum TC and LDL‐C levels when experienced 3 months of hypocaloric dietary intervention (de Luis et al. 2011; Deluis et al. 2010). It was also demonstrated that the A allele carriers of *FAAH* rs324420 had decreased TG and TC after 6 weeks low‐fat diets (Aberle et al. 2007). However, it was also reported that the decreases in TC and LDL‐C levels after 3 months of high polyunsaturated fat dietary intervention were not associated with *FAAH* rs324420 (de Luis et al. 2013). In the present study, we provided the evidence that the interactions occurred between *FAAH* rs324420 and dietary patterns to influence serum HDL‐C levels. Meanwhile, the dietary intake of fat affected the association of *FAAH* rs324420 with HDL‐C, which was gender‐dependent (Table 2, Figure 3). Particularly, the intake of fat and BMI were observed to be the predictors of serum HDL‐C levels in the male A allele carriers and the female CC homozygotes, but not in the male CC homozygotes and the female A allele carriers. We could not fully explicate the opposite effects of fat intake on HDL‐C in the female versus male participants categorized by *FAAH* rs324420 because limited literatures could be found at the current stage. Nevertheless, sex hormones could be among the reasonable explanations. For example, FAAH was found to be a contributor to the changes in plasma lipid profiles (Rahman et al. 2021) and regulated by estrogen (Maia et al. 2017). Taken together, our results reflect the complexity of ECS itself as well as its complex interactions with the diet and gender on lipid profiles (Alexander 2015) since *FAAH* rs324420 was closely interacted with FAAH protein expression and enzymatic activity (Chiang et al. 2004).

The present study also existed some limitations. First, the expressions of *FAAH* mRNA and concentrations of FAAH protein were not measured due to the intrinsic limitation of population studies. Second, in the present research, the dietary intake was gathered by dietary questionnaires based on 3‐day diet records. The dietary habits of our study population may not be accurately reflected in the data.

## 5 Conclusions

The results of our research indicate that the serum level of HDL‐C is positively associated with the fat intake and negatively with BMI in the male CC homozygotes and the female A allele carriers, but only negatively with BMI in the male A allele carriers and the female students with the CC genotype of *FAAH* rs324420 among Chinese Han adolescents, suggesting interactions of *FAAH* rs324420 with diets, BMI, and genders on the levels of HDL‐C. These results may contribute to provide a new perspective of precision dietary interventions for dyslipidemia. Biological backgrounds including gender, BMI, and *FAAH* rs324420 need to be taken into account when diets are used to prevent or treat low HDL‐C of hyperlipidemia, MetS, and cardiovascular disease in adolescents.

## Author Contributions

**Yi Lin Shen:** formal analysis (lead), investigation (lead), visualization (lead), writing – original draft (lead). **Li Qiu:** formal analysis (supporting), investigation (supporting). **Jia Jing Cai:** investigation (supporting), validation (supporting). **Qi Wei Guo:** investigation (supporting), validation (supporting). **Xu Chen:** investigation (supporting), validation (supporting). **Guo Ming Su:** investigation (supporting), validation (supporting). **Jia Lin:** project administration (lead), writing – review and editing (equal). **Ding Zhi Fang:** conceptualization (lead), data curation (lead), funding acquisition (lead), resources (lead), supervision (lead), writing – review and editing (equal).

## Conflicts of Interest

The authors declare no conflicts of interest.

## Data Availability

The interviews (datasets) generated and/or analyzed during the current study are not publicly available due to the information that could compromise the participant's privacy/consent but are available from the corresponding author upon reasonable request.
